# Vitamin D deficiency at the time of delivery – Prevalence and risk of postpartum infections

**DOI:** 10.1371/journal.pone.0226673

**Published:** 2019-12-19

**Authors:** Daniel Axelsson, Jan Brynhildsen, Marie Blomberg

**Affiliations:** 1 Department of Obstetrics and Gynecology, Ryhov County Hospital, Jönköping, Sweden and Department of Clinical and Experimental Medicine, Linköping University, Linköping, Sweden; 2 Department of Obstetrics and Gynecology, and Department of Clinical and Experimental Medicine, Linköping University, Linköping, Sweden; Copenhagen University Hospital Holbæk, DENMARK

## Abstract

**Background:**

Postpartum infections are a common cause of morbidity after childbirth. Vitamin D deficiency has been shown to increase the risk for several infections in a non-pregnant population. Vitamin D deficiency has been described as common in pregnant women.

**Objective:**

To investigate whether vitamin D deficiency in pregnant women in labor was associated with an increased risk of overall postpartum infectious morbidity within eight weeks of delivery. A secondary aim was to estimate the prevalence of vitamin D deficiency among pregnant women in Linköping, Sweden at the time of delivery.

**Material and methods:**

Serum vitamin D levels in labor were analyzed for 1397 women. Vitamin D deficiency was defined as serum levels <50 nmol/L. All ICD-10 codes given to the women eight weeks postpartum were reviewed and postpartum infections were defined as the presence of an ICD-10 code suggestive of infection. The prevalence of postpartum infections among women with sufficient vitamin D levels was compared with women with vitamin D deficiency. Adjusted Odds Ratios and 95% confidence intervals for postpartum infections were calculated using multivariate logistic regression analysis.

**Results:**

Fifty eight per cent of the women had serum vitamin D levels <50 nmol/L. The proportion of women with vitamin D deficiency varied, as expected, with season. No association between vitamin D deficiency and postpartum infections was found. For vitamin D 25–50 nmol/L the adjusted Odds Ratio was 0.85 (95% confidence interval 0.56–1.29) and for vitamin D <25 nmol/L the adjusted Odds Ratio was 1.15 (95% confidence interval 0.66–2.03). Women who smoked or who had a cesarean section had an increased risk of postpartum infections.

**Conclusions:**

Vitamin D deficiency was more common than previously reported in Swedish pregnant women. No association between vitamin D deficiency and postpartum infections was found. Other well-known risk factors for postpartum infection were identified.

## Introduction

Postpartum infections have been reported to affect 5–24% of women in Sweden, and 7.5% of delivered women are treated with antibiotics due to postpartum infections [1–3]. The most commonly reported postpartum infections are endometritis, urinary tract infection (UTI), wound infections (WI) and mastitis [1, 2].

The most studied obstetrical event associated with postpartum infection is cesarean section (CS) [4, 5]. Other identified risk factors are maternal diabetes mellitus, young maternal age, the presence of bacterial vaginosis during pregnancy, a high number of internal examinations, prolonged rupture of membranes, meconium stained amniotic fluid and postpartum hemorrhage [3, 6, 7]. Anemia at the time of delivery, or as a cause of obstetric events, is also a well-known risk factor for postpartum infections [6, 8].

There is a need to increase the knowledge of other predisposing conditions which are not primarily related to the pregnancy or delivery itself, in order to increase the opportunity for prevention.

Vitamin D plays an important role in the human immune defense against infections, and in epidemiological studies vitamin D deficiency has been shown to increase the risk for various infections in a non-pregnant population, including tuberculosis, HIV, respiratory tract infections, HCV infections and methicillin-resistant *Staphylococcus aureus* infection [9, 10]. Vitamin D supplementation has been shown to prevent acute respiratory infections [9, 11].

Vitamin D deficiency is usually defined as serum concentrations of the main circulating vitamin D, 25 hydroxy vitamin D (25OHD), below 50 nmol/L [12]. Vitamin D deficiency is common during pregnancy [10, 13, 14] and has been reported to be associated with bacterial vaginosis during pregnancy [15]. This is clinically relevant since the presence of bacterial vaginosis has been shown to increase the risk of postpartum infections [7, 11].

Thus, vitamin D deficiency has been correlated to an increased risk of several different infections, and such deficiency is also common in pregnant women. Consequently, pregnant women with vitamin D deficiency may have a higher risk of postpartum infections. The association between vitamin D deficiency in pregnant women and postpartum infections has not yet been studied.

The purpose of this study was to investigate whether vitamin D deficiency in pregnant women at the time of delivery is associated with an increased risk of overall postpartum infectious morbidity. The hypothesis was that vitamin D deficiency defined as serum 25OHD concentration <50 nmol/L is associated with a higher risk of postpartum infections. Furthermore, this study aimed to estimate the prevalence of vitamin D deficiency among these women.

## Materials and methods

The women included in this study were consecutively chosen from the local Pregnancy Bio bank (GRABB) at the Department of Obstetrics and Gynecology, Region Östergötland, Sweden. The purpose of this bio bank is to facilitate research on pregnancy-related topics. GRABB holds samples, obtained between 2011 and 2018, from nearly 8000 pregnant women. The women were asked to participate in the project at their first visit to the antenatal clinic during pregnancy weeks 6 to10. After providing informed written consent the women agreed to have blood samples collected during pregnancy (at 8 to 11 weeks and at 25 weeks) and from April 2014 also at delivery. Approximately 50% of all pregnant women agreed to participate and also gave consent for future use of their medical records for research purposes. At the time of the study, 25 percent of all included women also had samples drawn at the time of delivery (n = 1400). The delivery samples were drawn as soon as possible after the woman was admitted to the delivery ward and was in active labor.

Blood was collected in a test tube with a clot activator and gel for serum separation. One hour after sampling, the blood was centrifuged, aliquoted, and the serum was stored at -70 degrees Celsius in the local biobank (register number 185, at the department of Obstetrics and Gynecology, Östergötland County Council)

Serum 25OHD was analyzed using the standard analysis method (LIAISON^®^ 25 OH Vitamin D TOTAL Assay chemiluminescent immunoassay (CLIA)–DiaSorin Inc, USA) at the accredited laboratory at the Department of Clinical Chemistry, Linköping University Hospital (SWEDAC 1342). The analysis method, which measures 100% of D2 and D3 (OH) vitamin D, is certified according to the Vitamin D Standardization Program (VDSP) and is continuously validated against LC-MS/MS by an external quality control program (DEQAS, London, UK). According to the reference values of the method, vitamin D deficiency was defined as serum 25OHD <50 nmol/L. Serum concentrations below 25 nmol/L were classified as moderate deficiency, and values below 12.5 nmol/L were classified as severe deficiency. Toxic concentration of 25OHD was defined as >325 nmol/L [16].

Data on maternal characteristics, mode of delivery and postpartum hemorrhage were extracted for all included women from the obstetrical medical records (Obstetrix^®^, Cerner, USA).

An administrator at the Department of Obstetrics and Gynecology, Linköping University Hospital then obtained diagnoses from the same women’s medical records (Cosmic®, Cambio, Sweden), which contain records and diagnoses from all medical care providers in the County of Östergötland. For the purpose of this study, all diagnoses given up to eight weeks postpartum were extracted. The researchers then reviewed all diagnoses manually and women with one or several diagnoses suggestive of infection were considered as having had a postpartum infection. An *obstetric* infection was defined as the presence of an ICD-10 code indicating endometritis (O85.9), UTI (O86.2, N30 or N 10.9), infectious mastitis or breast abscess (BA) (O91 or N61) or WI (O86.0).

Based on previous studies we assumed that one third of the pregnant women would have serum levels of 25OHD <50 nmol/L and thus could be considered as having a vitamin D deficiency [13]. We hypothesized that vitamin D deficiency would be associated with a 50% increased risk of postpartum infection. To reveal this difference, a power analysis with alpha 0.05 and beta 0.80 showed that 1243 women needed to be included in the study. As the number of women in the bio bank with samples from the time of delivery only slightly exceeded this number, we decided to include all 1400 women.

Data were analyzed using SPSS Version 25. Descriptive statistics were presented as mean score, standard deviation, and absolute and relative frequency. Maternal characteristics were investigated using chi-squared tests for categorical variables and t-tests for numerical variables. Obstetric variables were entered in univariable and multivariable logistic regression analyses to evaluate the outcomes. Variables with a p-value <0.1 in the univariable analysis were considered as possible confounders and used in the multivariable analysis. Crude and adjusted odds ratios (ORs and aORs) were calculated with 95% confidence intervals (CI). All analyses were two-sided, and P-values less than 0.05 were considered as statistically significant.

The Regional Ethical Review Board in Linköping approved the study (2017/401-31)

## Results

**Fig 1:**
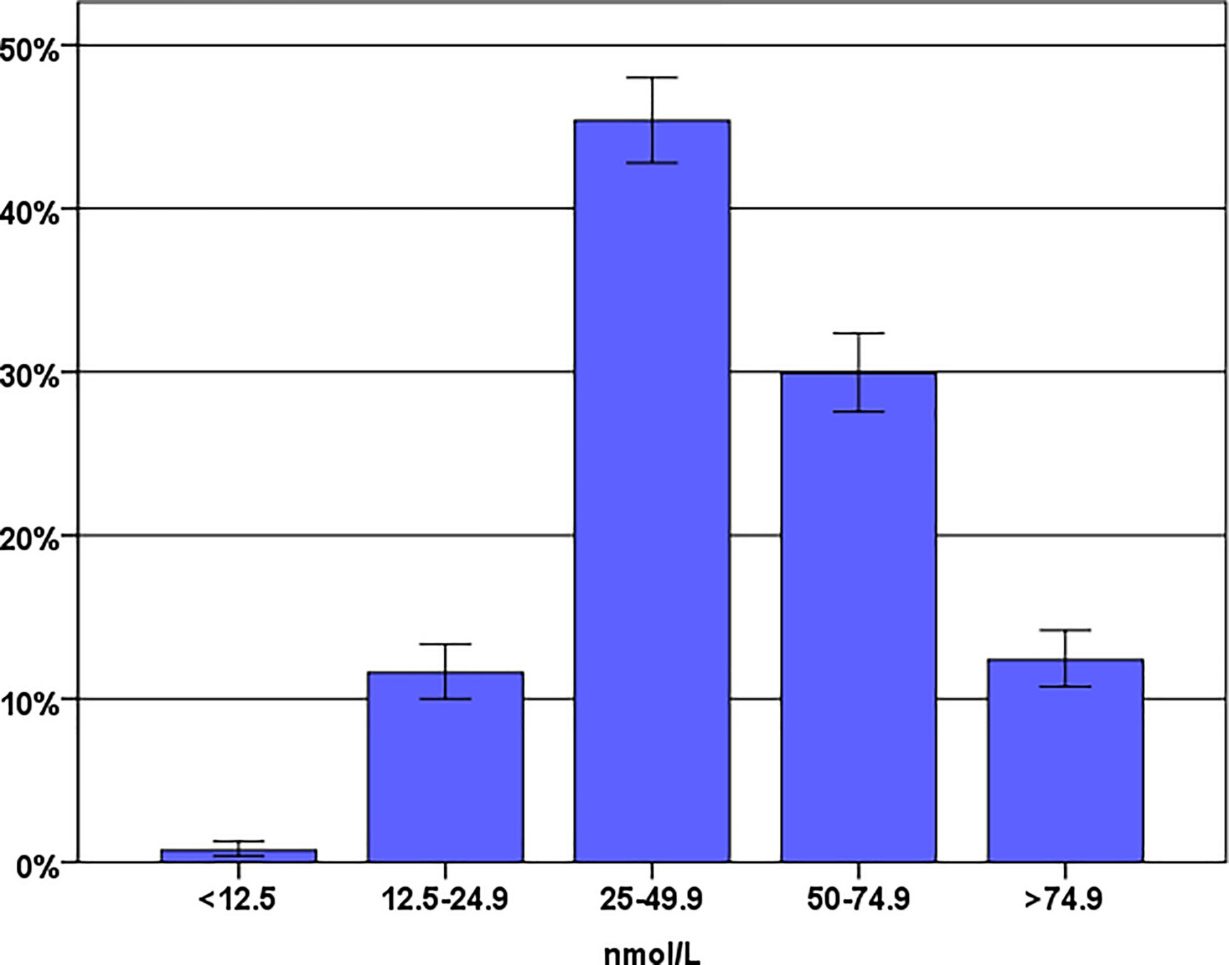
Distribution of serum 25OHD concentrations in pregnant women at the time of delivery.

Out of the 1400 samples taken from women in labor, one was excluded due to a registration error in the database and two were excluded since the test tube contained whole blood. The study population thus consisted of 1397 women with information on 25OHD concentrations in labor. A total number of 806 women (58%) had 25OHD concentrations below 50 nmol/L at the time of delivery, and were considered vitamin D deficient. Twelve percent (172/1397) had 25OHD concentrations below 25 nmol/L which is considered as moderate vitamin D deficiency. No toxic levels were identified (Fig 1). The mean serum 25OHD concentration was 45.3 nmol/L (SD 21.6), and the median was 48.3 nmol/L.

**Fig 2:**
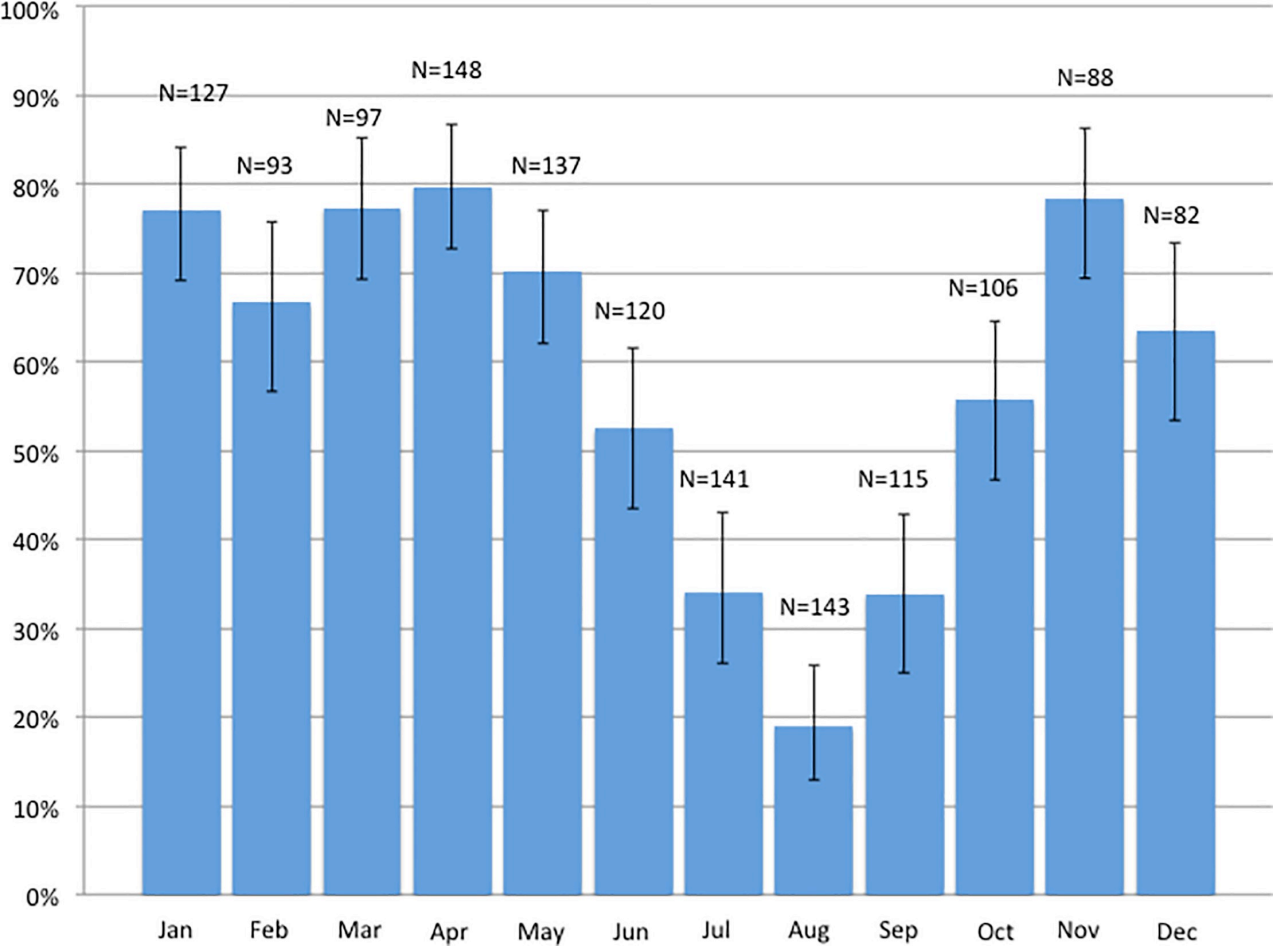
Proportion of pregnant women with vitamin D deficiency (<50 nmol/L) at the time of delivery according to month of sampling and total number (N) of samples collected per month.

The deliveries occurred evenly over the months during the study period (2014-04-01 to 2016-10-02). There was an expected variation over the year regarding the proportion of women with vitamin D deficiency (Fig 2).

Of the 1397 women in this study, 1227 (88%) had a vaginal, non-instrumental delivery, 71 (5.1%) were delivered by CS and 99 (7.1%) had a vacuum delivery.

**Fig 3:**
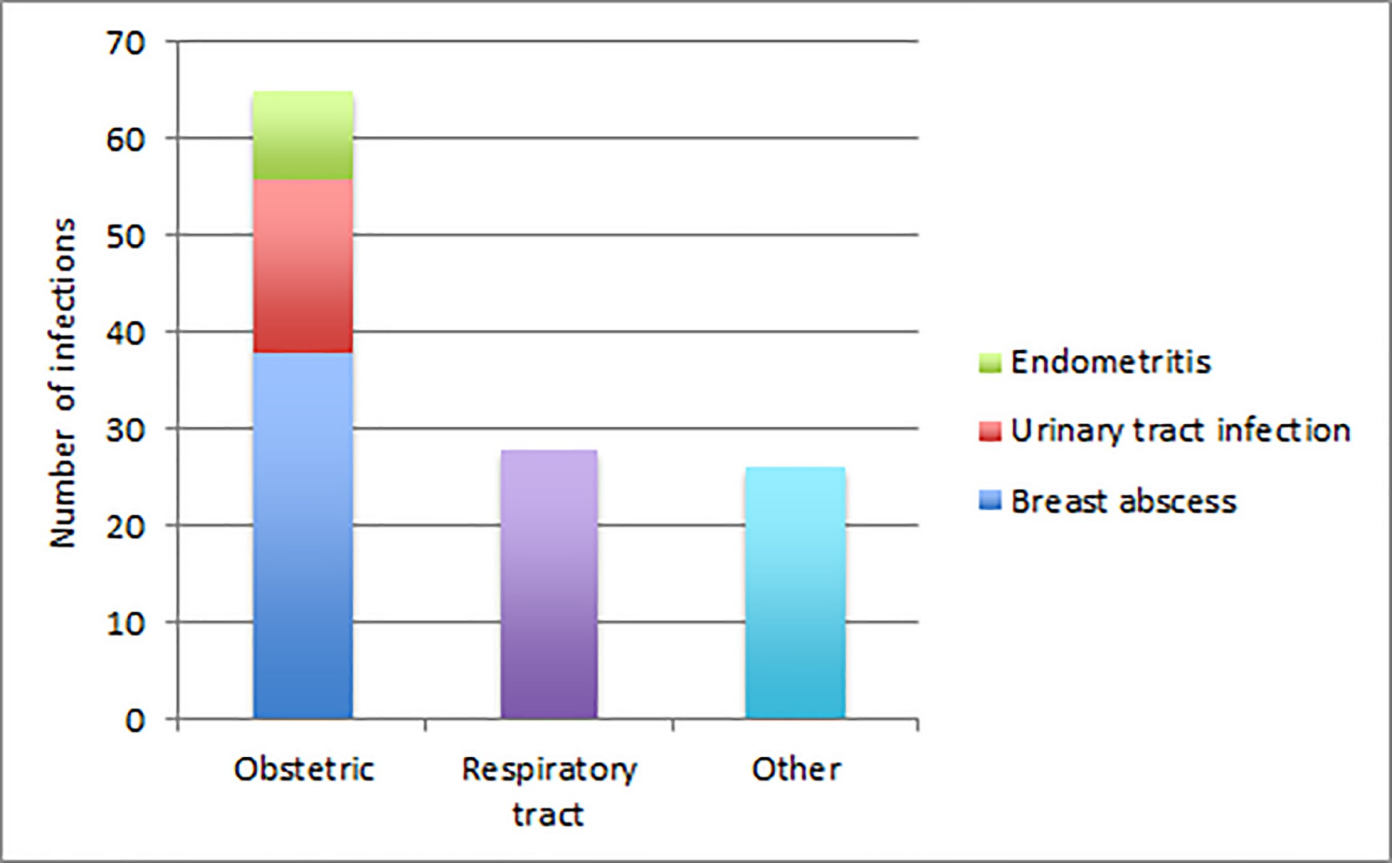
The distribution of infections, in absolute numbers, from delivery up to eight weeks postpartum.

A total number of 119 (9%) women had together 127 diagnoses suggestive of infection. Of these, 65 (5%) women had diagnoses suggestive of an obstetric infection (endometritis, UTI and mastitis). The distribution of the infections is presented in Fig 3.

Maternal and obstetric characteristics of the study population are presented in Table 1.

**Table 1:** Maternal and obstetric characteristics, and vitamin D categories of the study population.

	No infection N = 1278	Infection N = 119	p-value
Maternal age	N(%)	N(%)	0.934
<25	116 (9.1)	12 (10.1)	
25–29	449 (35.1)	39 (32.8)	
30–34	469 (36.7)	46 (38.7)	
>34	244 (19.1)	22 (18.5)	
Parity			0.045
Parous	640 (50.1)	71 (59.7)	
Primiparous	638 (49.9)	48 (40.3)	
Smoking in early pregnancy			0.039
No smoking	1253 (98.4)	114(95.8)	
Smoking	20 (1.6)	5 (4.2)	
Body Mass Index			0.423
<25	829 (65.3)	75 (63.0)	
25–29.9	318 (25.0)	28 (23.5)	
≥30	123 (9.7)	16 (13.4)	
Gestational age			0.458
<37 weeks	29 (2.3)	4 (3.4)	
≥37 weeks	1244 (97.7)	115 (96.6)	
Mode of delivery			0.009
Vaginal delivery	1220 (95.4)	107 (89.9)	
Cesarean section	59 (4.6)	12 (10.1)	
Postpartum hemorrhage			0.324
<1000 ml	1167 (93.7)	106 (91.4)	
≥1000 ml	78 (6.3)	10 (8.6)	
25OHD concentrations			0.333
≥50 nmol/L	539 (42.2)	52 (43.7)	
25–50 nmol/L	586 (45.9)	48 (40.3)	
<25 nmol/L	153 (12)	19 (16.0)	

Women with postpartum infection were more often smokers, had a previous childbirth and had been delivered by CS in the current pregnancy. Among women with no infection, 42% had a 25OHD concentration ≥50 nmol/L; the corresponding value in women with a diagnosed postpartum infection was 44%. The association between postpartum infection and mild or moderate vitamin D deficiency is shown in Table 2. There was no increased risk of postpartum infection whether the woman had mild (OR 0.94 95%CI 0.64–1.38) or moderate (OR 1.40 95%CI 0.83–2.35) vitamin D deficiency.

**Table 2:** Demographic factors, vitamin D status and putative risk factors for postpartum infections.

	Odds Ratio (95%CI)	Adjusted odds ratio* (95%CI)
25OHD concentrations		
≥50 nmol/L	ref	ref
25–50 nmol/L	0.85 (0.56–1.28)	0.85 (0.56–1.28)
<25 nmol/L	1.29 (0.74–2.24)	1.15 (0.66–2.03)
Maternal age		
<25	1.19 (0.60–2.35)	
25–29	ref	
30–34	1.13 (0.72–1.76)	
>34	1.04 (0.60–1.79)	
Parity		
Parous	ref	
Primiparous	0.68 (0.46–0.99)	
Smoking in early pregnancy		
No smoking	ref	
Smoking	2.75 (1.01–7.46)	
Body Mass Index		
<25	ref	
25–29.9	0.97 (0.62–1.5)	
≥30	1.45 (0.81–2.55)	
Gestational age		
<37 weeks	1.49 (0.52–4.32)	
≥37 weeks	ref	
Mode of delivery		
Vaginal delivery	ref	
Cesarean section	2.32 (1.21–4.45)	
Postpartum hemorrhage		
<1000 ml	ref	
≥1000 ml	1.41 (0.71–2.81)	

*Adjustments were made for parity, smoking and mode of delivery.

Odds of postpartum infection were higher among women who smoked (OR = 2.75 95%CI 1.01–7.46) and delivered by CS (OR = 2.32 95%CI 1.21–4.45) and lower among primiparous women (OR = 0.68 95%CI 0.46–0.99). Adjustment for these factors did not change the risk estimates for moderate and mild vitamin D deficiency in relation to postpartum infection.

## Discussion

Almost six out of ten women (58%) in labor had 25OHD concentrations below 50 nmol/L and more than 12% had serum concentrations less than 25 nmol/L. The proportion of women with vitamin D deficiency varied with sampling season, with the lowest proportions during summer months. Moderate and mild vitamin D deficiency was more common than has been previously reported in pregnant Swedish women [13, 14].

We were not able to identify vitamin D status during pregnancy as a predictor of postpartum infections. Instead, well-known risk factors such as smoking and CS were found to be significant risk factors for postpartum infections. This is in accordance with several previous studies [5, 8].

Previous studies on infections related to vitamin D deficiency have predominantly focused on TBC, HCV, HIV, methicillin-resistant *Staphylococcus aureus*, and influenza, which are rare in a Swedish pregnant population [9]. It could be speculated that the obstetric infections in the present population were more directly related to the delivery and obstetric interventions. A possible effect of vitamin D deficiency on the susceptibility for infections may thus have been overshadowed by the delivery itself, obstetric interventions performed, and breast feeding.

Infections were solely defined as the presence of ICD-10 codes suggestive of infection. These data were retrospective and dependent on individual doctors’ diagnoses and registration in the woman´s medical records. No data on prescriptions of antibiotics, which could have been used as a proxy for infection were reviewed. A majority (55%) of the infections were obstetric infections, with BA as the most common. This is in accordance with results from previous studies [2, 3]. The overall prevalence of postpartum infections was however lower than previously reported [2]. This may imply a selection bias in the population studied.

Surprisingly, we found no diagnoses of WI. WI have been reported to affect 1.2% of puerperal women in Sweden, and a certain number of WI should have been expected [2]. This may at least partly be explained by a low number of CS in this cohort as CS is a major cause of WI [8]. Moreover, WI may have been misclassified as “other infection”

In the present study we lacked information about ethnicity among the women included. With the present study design, information about ethnicity was not possible to obtain, since this data is not available in the medical records. Based on the fact that good ability in reading and understanding the Swedish language was needed for consent and inclusion in the biobank, we can assume that the study population may have been skewed and possibly included a low risk population, both regarding infection and vitamin D deficiency. The assumption that the risk of infection in the study population could be lower than that of the general Swedish population could also be supported by the fact that only 5.1% of the women in the study population were delivered by cesarean section, whereas this figure for Sweden in general was 17% in 2015 [17]. Corresponding figures for Swedish women regarding smoking in early pregnancy was 5.2% in 2015 and the proportion of women in Sweden with BMI >25 the same year was 39% [17]. However, this probably does not affect the possibility to extrapolate the results regarding the non-association between vitamin D deficiency and postpartum infections to the entire population.

The prevalence of vitamin D deficiency in the present study was considerably higher than previously reported in pregnant Swedish women. A previous Swedish study (13) found that one third of pregnant women in the northern part of Sweden had serum concentrations of 25OHD below 50 nmol/L in the third trimester [13]. The differences between the studies in reported 25OHD concentrations cannot be explained by differences in time of the year, vitamin D supplementation, or differences in guidelines as the samplings were performed during the same years and the dietary supplementation and national advice for vitamin D supplementation during pregnancy were the same. The sample sizes differ considerably between the study by Lundqvist et al (13) and the present study, which may play a role in the difference. Another possible explanation to the difference could be that different assay methods for 25OHD were used. In the study from north of Sweden (13) the LC-MS/MS method was used whereas the analyses in the present study were performed with the DiaSorin Liasion XL method. In the DEQAS review 2016–2017 the mean % bias from NIST assigned values ware presented for several different assay methods. DiaSorin Liasion XL and LC-MS had approximately the same biases during the study period, with DiaSorin around -5 to -10% and LC-MS around +5 to +10%. With that difference being consistent, it is possible that a higher proportion of women would be considered as vitamin D deficient when the DiaSorin Liasion XL method is used, compared to the LC-MS/MS method, when the same cut-off values are used. Hence, the choice of analysis method might affect the results, which should encourage one to be cautious when comparing studies performed with different assay methods.

Other studies from southern Sweden and Denmark, have reported that 25–42% of pregnant women had vitamin D deficiency in the first trimester[14, 18–20]. Two of these studies reported that serum concentrations of 25OHD increased by almost 10 nmol/L from the first to third trimesters, suggesting that an even higher number of the women in the present study could have been vitamin D-deficient in early pregnancy. With 58% of women having low levels of vitamin D, the cut-off value for deficiency can be questioned and maybe the reference values have to be adjusted in a pregnant population. On the other hand, there is consensus about levels of >50nmol/L being important for maximum bone health, with some researchers believing that even higher concentrations are required [21, 22].

## Conclusions

Vitamin D deficiency, as defined by serum 25OHD less than 50 nmol/L, may be more common among pregnant women than has previously been reported, but was not associated with an increased risk of overall postpartum infectious morbidity, nor with an increased risk of postpartum obstetric infections. The same conclusion was valid for women with vitamin D levels below 25 nmol/L. Further efforts should be made to improve vitamin D status in pregnancy.

## Supporting information

S1 Supplement Data sheet with 25OHD results, maternal characteristics and diagnoses. (XLSX)
